# Supraventricular Tachycardia Ablation and Its Effects on Anxiety Medications

**DOI:** 10.7759/cureus.24609

**Published:** 2022-04-29

**Authors:** Mohamed Mahmoud, Justin Haloot, Khadija El Kortbi, Vanessa Rodriguez Fuenmayor, Mubeen Cheema, Auroa Badin

**Affiliations:** 1 Internal Medicine, University of Texas Health Science Center at San Antonio, San Antonio, USA; 2 General Practice, Hassan II University, Faculty of Medicine, Casablanca, MAR; 3 Cardiovascular Disease, University of Texas Health Science Center at San Antonio, San Antonio, USA; 4 Cardiac Electrophysiology, Riverside Methodist Hospital, Columbus, USA

**Keywords:** paroxysmal supraventricular tachycardia, cardiac electrophysiology, snri, ssri, psychiatric medications, svt ablation, psvt, catheter ablation, avnrt, anxiety

## Abstract

Background: Patients with true paroxysmal supraventricular tachycardia (PSVT) are frequently misdiagnosed with panic or anxiety disorders due to similar symptoms of palpitations, light-headedness, dyspnea, or chest discomfort. Unrecognized PSVT can lead to unnecessary management with anxiety medications. Treatment of PSVT with catheter ablation may lead to a reduction in anxiety medications.

Methods: A total of 175 patients underwent successful PSVT ablation between January 1, 2010 and December 31, 2020. We examined symptoms at presentation, psychiatric medications prior to PSVT ablation, comorbidities, and psychiatric medications at three months post-ablation.

Results: Fifteen percent of patients who underwent successful PSVT ablation were being treated with psychiatric medications and included in the final study population. The most common symptoms were palpitations (80.77%), followed by dizziness (42.31%), and shortness of breath (34.62%). The average number of medications prior to ablation was 1.42 and decreased to 1.08 at three months post-ablation (p = 0.04). The average number of selective serotonin reuptake inhibitors (SSRIs), serotonin and norepinephrine reuptake inhibitors (SNRIs), and other anxiolytics also decreased but was not statistically significant.

Conclusion: In patients with anxiety and PSVT, catheter ablation reduced the average number of psychiatric medications.

## Introduction

Paroxysmal supraventricular tachycardia (PSVT) describes tachycardias with abrupt onset and termination of regular tachycardias originating above the bundle of His. This includes atrial tachycardia, atrioventricular nodal reentry tachycardia (AVNRT), and atrioventricular reentrant tachycardia (AVRT) [1]. The prevalence of PSVT has been reported to be approximately 2.29 per 1000 persons and an incidence of 36 per 100,000 persons per year. Symptoms of PSVT vary and include palpitations, shortness of breath, fatigue, chest pain, near syncope, diaphoresis, and anxiety [2].

PSVT has been associated with anxiety and panic disorders. This association may be due to the sudden onset of tachycardia, causing increased anxiety and stress levels [3-5]. However, the symptoms of PSVT appear to mimic anxiety and may offer another etiology for this relationship. One study found that 67% of patients with PSVT could be diagnosed with panic disorder based on the Diagnostic and Statistical Manual of Mental Disorders, Fourth Edition (DSM-IV) criteria [6]. In the same study, it was found that physicians attributed the symptoms of PSVT to panic, anxiety, or stress in 54% of patients [6]. Multiple studies have found that catheter ablation for PSVT decreased stress and anxiety levels [7-10]. However, these studies utilized symptomatic scoring systems such as the World Health Organization Quality of Life Brief Version (WHOQOL-BRIEF) domain scores and the Short Form health questionnaire (SF-36). To date, there has not been a study examining the effects of PSVT ablation on anxiety-related medication usage.

This study aims to examine the effect of catheter ablation on psychiatric medication utilization in patients with PSVT. We hypothesize that there will be a decrease in the number of psychiatric medications utilized post-ablation.

This article was previously posted to the Authorea preprint server on November 29, 2021.

## Materials and methods

Data source 

This is a retrospective analysis of all patients who underwent catheter ablation for PSVT between January 1, 2010 and December 31, 2020. A research protocol was designed and approved by the Institutional Review Board (IRB) at the University of Texas Health Science Center at San Antonio (approval number 20200567EX). Medical records were manually reviewed, and data were collected by the research team. 

Patient selection

Patients who underwent catheter ablation for PSVT from January 1, 2010 to December 31, 2020, were examined. Psychiatric medications prescribed to the patient prior to ablation were recorded, and symptoms of PSVT were also noted. Patients that were not on any psychiatric medications and patients with unsuccessful ablation were excluded. Of the included patients, the number of psychiatric medications at three months post-ablation was noted. Baseline characteristics including age, ethnicity, gender, and cardiovascular medications were recorded. 

Outcome measures

The primary outcome was the number of psychiatric medications for PSVT patients pre- and three months post-ablation. Patients that were prescribed psychiatric medications were followed to examine the difference in the number of medications at three months post-ablation. Medications evaluated were selective serotonin reuptake inhibitors (SSRIs), serotonin and norepinephrine reuptake inhibitors (SNRIs), benzodiazepines (BZDs), anxiolytics, antipsychotics, tricyclic antidepressants, buspirone, trazodone, and bupropion. 

Statistical methods

The mean numbers of psychiatric medications, SSRIs, SNRIs, BZDs, other anxiolytics, and antipsychotics were analyzed for differences based on the paired samples t-test. P-values less than 0.05 were regarded as statistically significant.

## Results

Between January 1, 2010 and December 31, 2020, 175 patients underwent SVT ablation. Fifteen percent of these patients were being treated with psychiatric medications and included in the final study population. Patient characteristics can be found in Table 1. The average age was 50.08 + 17.92 years. 57.7% were female and 26.9% had been diagnosed with hypertension. 65.4% of PSVT patients taking psychiatric medications had a formal diagnosis of generalized anxiety disorder. In terms of medical management, 46.2% of these patients were on beta-blockers, 3.9% on calcium channel blockers, 15.4% on antiarrhythmic medications, and 3.9% on direct oral anticoagulants (DOACs).

**Table 1:** Demographics of paroxysmal supraventricular tachycardia (PSVT) patients taking psychiatric medications.

Gender	
Male (n, %)	11 (42.3%)
Female (n, %)	15 (57.7%)
Race	
Caucasian (n, %)	15 (57.7%)
Hispanic (n, %)	8 (30.8%)
African American (n, %)	0 (0%)
Asian American/ Pacific Islander (n, %)	3 (11.5%)
Other	0 (0%)
Medical History	
Hypertension (n, %)	7 (26.9%)
Asthma (n, %)	8 (11.5%)
Chronic Obstructive Pulmonary Disease (n, %)	1 (3.9%)
Anxiety (n, %)	17 (65.4%)
Other Medications Prior to Ablation	
Beta Blockers (n, %)	12 (46.2%)
Calcium Channel Blockers (n, %)	1 (3.9%)
Anti-Arrhythmic Drugs (n, %)	4 (15.4%)
Direct Oral Anticoagulants (n, %)	1 (3.9%)

**Figure 1:**
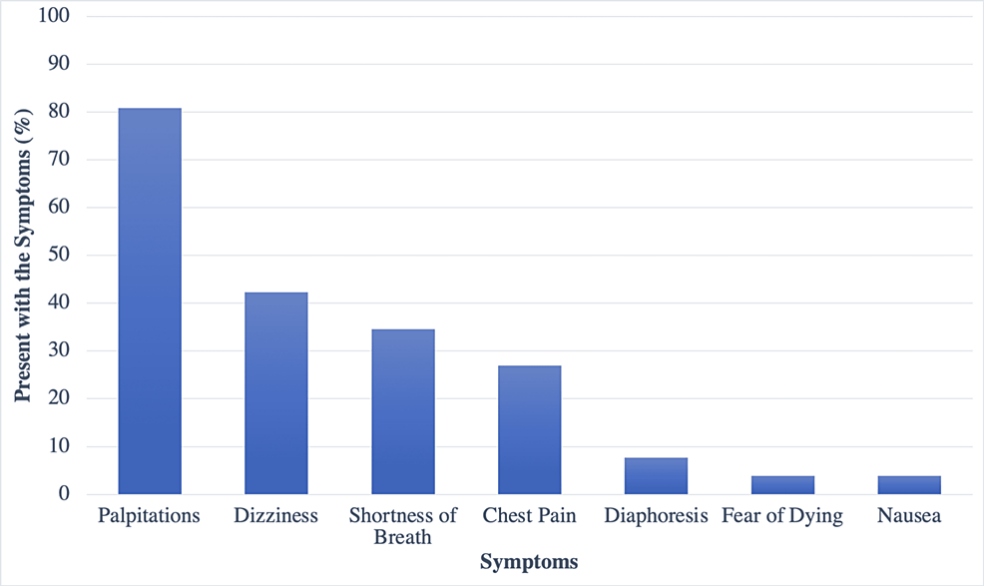
Most common symptoms of patients with paroxysmal supraventricular tachycardia (PSVT) and prescribed psychiatric medications.

With regards to the presentation of PSVT, the most common symptoms reported were palpitations (80.77%), followed by dizziness (42.31%) and shortness of breath (34.62%) (Figure 1). 

Psychiatric medications

The average number of psychiatric medications prior to ablation was 1.42 and was reduced at three months post-ablation to 1.08 (p-value 0.04). When broken by category, the average SSRI number prior to ablation was 0.54, and at three months post-ablation it was 0.42 (p-value 0.18). The average number of SNRIs also decreased from 0.19 to 0.15 (p-value 0.33). The average number of anxiolytics also decreased from 0.31 to 0.19 (p-value 0.18). Finally, the number of antipsychotics remained stable with an average of 0.12 prior to ablation and 0.15 at three months post-ablation (p-value 0.57) (Table 2). 

**Table 2:** Psychiatric medications of paroxysmal supraventricular tachycardia (PSVT) patients. SSRI: selective serotonin reuptake inhibitors, SNRI: serotonin and norepinephrine reuptake inhibitors

	Pre-ablation	3 months post-ablation	P-value
Number of Psychiatric Medications	1.42	1.08	0.04
Number of SSRIs	0.54	0.42	0.18
Number of SNRIs	0.19	0.15	0.33
Number of Other Anxiolytics	0.31	0.19	0.18
Number of Antipsychotics	0.12	0.15	0.57

## Discussion

The main findings of this study demonstrate that catheter ablation can lead to a decrease in total psychiatric medications in PSVT patients that were being treated with psychiatric medications. Previous studies demonstrated that ablation improves subjective symptoms through scoring systems [7,8,10]. This is the first study that provides objective evidence that psychiatric medications significantly decrease in these patients.

PSVT is defined as a clinical syndrome noted by the presence of regular and rapid tachycardia [1]. Palpitations, dyspnea, hyperventilation, syncope, sweating, chest pain, and anxiety are the most reported symptoms [2]. Our study demonstrated similar presentations, with the most common complaint being palpitations. In a previous study, 67% of patients with PSVT had symptoms consistent with the diagnosis of panic disorder based on DSM-IV criteria [6]. The criteria remain similar based on the updated DSM-V; therefore, PSVT could continue to remain misdiagnosed as a panic or anxiety disorder [11].

It has been reported that anxiety may be exacerbated by PSVT episodes. The sudden onset of PSVT can trigger anxiety disorders in patients [3]. One study reported that anxiety disorder has been present in approximately 25% of patients with PSVT; interestingly, the same study reported that PSVT was unrecognized over 50% of the time [6]. As anxiety levels increase, patients are at an increased risk for further PSVT [5,9]. Therefore, patients can often get caught between anxiety and panic attacks between episodes of PSVT. This confounds the clinical picture, leading to misdiagnosis, incorrect management, and potentially escalating anti-anxiety medications.

Catheter ablation is a Class 1 recommendation for the treatment of PSVT based on the 2015 American College of Cardiology/American Heart Association/ Heart Rhythm Society guidelines [1]. Ablation has a high success rate, greater than 93%, dependent on the mechanism [12,13] with a minimal complication rate [12]. It is highly effective for eliminating further episodes of PSVT [14,15] and has been proven to be cost-effective when compared to chronic antiarrhythmic drug therapy [8,16-19]. However, there has been less evidence on the effects of ablation on anxiety in patients with PSVT. Current studies utilized subjective scoring systems to demonstrate improvement in these psychiatric disorders. Yildrim et al. used the WHOQOL-BREF domain scores [8,20] and demonstrated a statistically significant improvement post-ablation. Papiashvilli et al. used the State and Trait Anxiety Inventory (STATI) and found that PSVT patients had improved situational and general anxiety levels [7,21]. The same group also utilized the SF-36 questionnaire and found significant improvement in physical, social, and emotional health scores after ablation [10,22]. Our study further supports these data by providing further objective evidence of the effect of PSVT ablation on anxiety disorders. 

We examined the number of medications and found a significant decrease in the number of psychiatric medications. We also examined different types of medications including SSRIs, SNRIs, antipsychotics, and other anxiolytics such as BZDs, bupropion, buspirone, and trazodone. These medications were chosen due to the current medical management of anxiety and panic disorders [23,24]. SSRIs and SNRIs are often the first-line treatment for anxiety. Typically, one agent is chosen and titrated up to the maximum dose. If the medication does not provide the desired outcome, the patient is switched to another agent, and the process is repeated. Refractory cases will be supplemented with other anxiolytics or BZDs as needed [25]. If the anxiety is uncontrolled with the previous process, antipsychotics may be utilized [26]. Therefore, we wanted to look at the total number of psychiatric medications as well as each individual subtype as a measure of anxiety disorder severity. For patients with uncontrolled anxiety, we expect the number of psychiatric medications to increase based on current guidelines. We found that patients with PSVT who were being treated for anxiety had a decrease in the total number of psychiatric medications. We also found that each subgroup had a decrease in the number of medications, but these were not statistically significant. This may be due to the limitation of a small sample size.

The main limitations of our study were the sample size and the relatively short period of follow-up. Other modes of managing anxiety such as cognitive therapy exist but were not recorded [27]. Further studies that can provide longer follow-up data and a larger sample size are required to make the results even more relevant.

## Conclusions

Patients with paroxysmal supraventricular tachycardia (PSVT) are frequently misdiagnosed with panic or anxiety disorders due to similar symptoms of palpitations, light-headedness, dyspnea, or chest discomfort. Unrecognized PSVT can lead to unnecessary management with anxiety medications. Previous studies demonstrated that ablation improves subjective symptoms through scoring systems. Ours is the first study that provides objective evidence that in patients with anxiety disorder and PSVT, the average number of psychiatry medications was reduced post catheter ablation.
